# A Data-Centric Augmentation Approach for Disturbed Sensor Image Segmentation

**DOI:** 10.3390/jimaging7100206

**Published:** 2021-10-06

**Authors:** Andreas Roth, Konstantin Wüstefeld, Frank Weichert

**Affiliations:** Department of Computer Science, TU Dortmund University, 44227 Dortmund, Germany; andreas.roth@tu-dortmund.de (A.R.); frank.weichert@tu-dortmund.de (F.W.)

**Keywords:** data augmentation, imaging artifacts, sensor images, deep learning, generative adversarial network

## Abstract

In the context of sensor-based data analysis, the compensation of image artifacts is a challenge. When the structures of interest are not clearly visible in an image, algorithms that can cope with artifacts are crucial for obtaining the desired information. Thereby, the high variation of artifacts, the combination of different types of artifacts, and their similarity to signals of interest are specific issues that have to be considered in the analysis. Despite the high generalization capability of deep learning-based approaches, their recent success was driven by the availability of large amounts of labeled data. Therefore, the provision of comprehensive labeled image data with different characteristics of image artifacts is of importance. At the same time, applying deep neural networks to problems with low availability of labeled data remains a challenge. This work presents a data-centric augmentation approach based on generative adversarial networks that augments the existing labeled data with synthetic artifacts generated from data not present in the training set. In our experiments, this augmentation leads to a more robust generalization in segmentation. Our method does not need additional labeling and does not lead to additional memory or time consumption during inference. Further, we find it to be more effective than comparable augmentations based on procedurally generated artifacts and the direct use of real artifacts. Building upon the improved segmentation results, we observe that our approach leads to improvements of 22% in the F1-score for an evaluated detection problem. Having achieved these results with an example sensor, we expect increased robustness against artifacts in future applications.

## 1. Introduction

A key goal of image analysis is to automatically extract information contained in an image using a suitable algorithm [1]. The devices used for image acquisition are usually based on either charge-coupled device (CCD) sensors [2] or complementary metal–oxide–semiconductor (CMOS) sensors [3]. Although the specific properties of recording techniques differ, all types induce artifacts caused by the process of capturing images [4].

We refer to all image signal components as artifacts that are not intended to be part of an image. These artifacts impede an automatic or human evaluation of recorded images, especially when they are similar to signals of interest, which can cause them to be falsely recognized as such. Artifacts should compromise the analysis of images as little as possible. Therefore, methods to reduce the influence of artifacts on an image are of particular interest [5]. The effects causing artifacts are called disturbances. These include, for example, instabilities of the used recording devices and other connected electronics, environmental influence, or flaws in the preprocessing software.

Artifacts are visually recognizable in a variety of shapes and intensities. Table 1 shows common artifact types occurring in sensor images, their sources, and algorithmic example methods which can be used to reduce these artifacts. The set of example artifacts can be divided into correlated and uncorrelated signals. Uncorrelated artifacts, also called random noises, are characterized by the absence of clear, detectable structures. Often, they originate from the sensor instruments themselves due to electronic instabilities or environmental influence [4,6,11]. Artifacts that show recognizable structures in the temporal, the spatial, or both dimensions are referred to as correlated. In distinction to random noise, these are also called structured noise [40,41]. In terms of their temporal behavior, most of the correlated and the uncorrelated artifacts are temporally changing, making them difficult to detect and reduce. Besides the determined differences of artifact types, it is worth noting that in practice, a signal does not only contain a single type of artifact but combinations of them.

Image-related tasks like classification, segmentation, and object detection are increasingly solved using deep learning [42,43,44]. This holds, in particular, for the field of sensor imaging. Examples include astronomical imaging [45], autonomous driving [46], fluorescence microscopy [47], X-ray [48], magnetic resonance (MR) [49], computed tomography (CT) [50], and histological imaging [51]. While access to an arbitrarily large amount of data could be used to form all possible combinations of signals of interest and artifact signals during training, a common problem is the limited availability of data, particularly in medical imaging tasks [52]. It is caused by high time and material costs for recording examples and intensified by data privacy restrictions that create further hurdles for the data collection [52]. Additionally, the annotation of images can be a time-consuming task requiring experts’ review [52]. For deep learning methods in sensor image analysis, it is therefore particularly desirable to develop approaches that deal with very limited data availability during the training stage.

**Figure 1:**
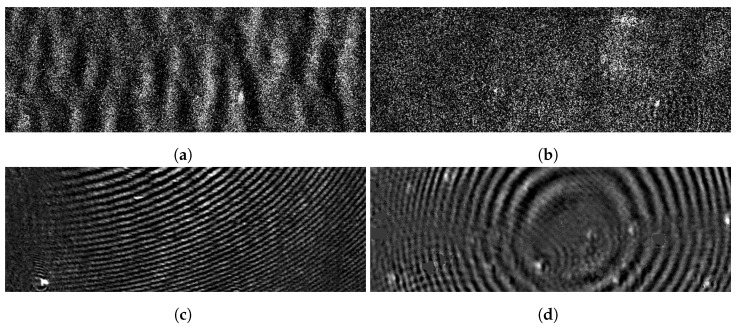
Example images extracted from different datasets recorded with the Plasmon-Assisted Microscopy of Nano-Objects (PAMONO) sensor after preprocessing and the application of dynamic contrast enhancement. Different dominating types of artifacts presented in Table 1 can be perceived. Random noise artifacts are present in each recorded image but vary in their intensities with differing environmental influences. (**a**) Washed out line artifact. (**b**) Dominant background noise with temporal brightness inconsistencies in an image region on the right. (**c**) Dominating higher frequency wave artifact with a center near the visible region. (**d**) Dominating lower frequency wave artifact with visible origin.

As an example of a sensor affected by different disturbances, Section 3 describes the Plasmon-Assisted Microscopy of Nano-Objects (PAMONO) sensor, which has been the subject of several research questions [53,54,55,56] and served as a starting point for the research presented in this paper. It is affected by disturbances during image acquisition, resulting in varying artifact characteristics, for which some are shown in Figure 1. Therefore, it offers a well-suited data basis to evaluate methods for increased robustness against artifacts.

Motivated by the observation above, we propose a data-centric approach that aims at increasing the robustness of learning methods against image artifacts. We use the term data-centric to describe that only training data is modified to maximize the performance of a learning procedure. At the same time, the existing model does not change. There is no deceleration or change in memory requirements during inference as only the learned weights are adjusted. We present an approach based on generative adversarial networks (GANs) [57], which overlays images with realistic but synthetically generated artifacts during the training of a segmentation network. The GAN is trained with real images containing only artifacts and learns to generate an arbitrary number of new artifact images. We do not need additional annotations for our approach. As an example for our method, we evaluate our GAN approach on PAMONO sensor data. We find that the effect of artifacts on a segmentation task is reduced significantly. We also show that the GAN approach is superior to alternative, non-learning approaches in the evaluated segmentation task. For comparison, we employ a procedural generation of combined wave artifacts based on qualitative observations and the direct use of real artifact images from recorded datasets.

The structure of this paper is as follows. Section 2 mentions related methods for reducing artifacts in image signals and popular methods for generating synthetic images. Section 3 details the PAMONO sensor and its recorded data as the basis for evaluating the presented approaches. Section 4.1 describes our approach for an overlay composed of realistic but synthetic artifact patterns utilizing the StyleGAN2-ADA [58] architecture. For direct comparison, Section 4.2 and Section 4.3 present methods for overlaying training images with real artifacts and the procedural generation of combined waves, respectively. We present the integration of our approach into experiments and the considered metrics in Section 5. The results are compared and discussed in Section 6. In the end, we give suggestions for future work in Section 7.

## 2. State of the Art

For the task of artifact reduction, examples for methods related to specific types of artifacts can be found in Table 1. It includes traditional as well as machine learning approaches. An overview focusing particularly on deep learning-based methods for image artifact removal is provided by Tian et al. [9]. It covers a wide range of approaches and structures them based on their methodological similarities. There are various traditional approaches such as Gaussian, median and bilateral filters [7,8], homomorphic filtering [13], methods based on physical models [25], morphological filters [30], Fourier- and wavelet-based filtering [10]. An early application of convolutional networks for image denoising was published by Jain and Seung [59]. The proposed strategy introduced a specific artifact removal network that outputs a clean image with reduced artifacts [59]. Since this learning strategy demonstrated its potential to reduce various artifacts, further work has followed this approach [60,61,62]. Disadvantages of these methods include an introduction of additional computational costs, additional memory requirements, and in some cases, the need for clean images without artifacts.

A different approach improves the robustness of an existing model against artifacts using augmentation methods [63]. The related methods are applied to an existing model by modifying or expanding training data during the optimization process. Since these methods only change data but not architectures, we refer to them as data-centric. This characteristic has the advantage that the methods can be applied during training and do not require the modification of an existing algorithm. Various methods for augmentation show drawbacks making them undesirable as they focus on uncorrelated noise [63], assume perfect artifacts [15], or rely on hand-crafted definitions for creating correlated artifacts [64]. In addition, reference images are rarely exploited. Reference images can be acquired without objects of interest and therefore contain only background and artifacts. They contain valuable information, especially for tasks with low data availability. We developed our approach to address these shortcomings. We exploit reference images and use both correlated and uncorrelated artifacts.

Cubuk et al. [65] proposed AutoAugment, a method to learn sequences of augmentations from a set of parametrized operations to improve the training process for an underlying network. As our approach is comparable to an augmentation operation within AutoAugment, the methods do not form alternatives but are combinable.

For tasks with low availability of labeled training data, various approaches augment existing data with synthetic images using GANs [66,67,68,69,70]. For example, Frid-Adar et al. [66] use a GAN to synthesize new images for CT scan data of liver lesions. Han et al. [67] follow a similar objective by generating synthetic brain MR images. Sandfort et al. [68] employ a CycleGAN [71] to expand a dataset of CT scans with synthetic images. Hee et al. [69] use a conditional GAN to generate brain metastases at desired locations in synthetic MR images. The mentioned methods do not use reference images but only images containing signals of interest. In contrast to that, our approach also uses reference images to take advantage of this information.

For GANs, as state of the art for image synthesis, recent developments show that they can be trained even with very limited amounts of data [58]. Driven by these findings, we make use of a StyleGAN2-ADA network [58] to generate realistic artifacts, which we use for the augmentation of existing training data.

## 3. PAMONO Sensor Image Streams

The following explanations characterize the images recorded with the Plasmon-Assisted Microscopy of Nano-Objects (PAMONO) sensor [53]. Since each recording of the device shows different types of dominant artifacts, this data serves as the basis for our evaluation.

The PAMONO sensor employs the effect of surface plasmon resonance (SPR) [72] to make individual nanoparticles visible as bright spots on preprocessed images. These spots become more difficult to detect with an increasing quantity or intensity of artifacts in the images. This functionality enables the use as a rapid test for the presence of viruses and virus-like particles (VLPs) and for counting nanoparticles in a sample [73]. The sensor visualizes particles of interest using a gold foil with an antibody coating on one side. The foil is attached to a flow cell containing a liquid sample, while the opposite side reflects a laser beam directed towards it. When specific particles in a sample attach to the antibody coating, the reflective properties of the gold foil change at this region, and the particles become visible in the reflected signal. This setup provides indirect imaging for the downstream detection of nano-sized objects. Further explanations of the technical aspects and application scenarios, such as detecting viruses, can be found in the literature [53,54,55,56]. While a high degree of reliability is essential for detecting nanoparticles, recording with the PAMONO sensor is prone to disturbances originating from its high sensitivity to changes in the nanometer scale, temperature dependence, sensitivity to external impacts, and contaminations of the analyzed samples [74]. This results in random noises originating from the electronics and the environment, wave and line artifacts resulting from air bubbles and dirt particles in a sample, and significant global and local brightness differences due to environmental changes or the preprocessing. In addition, local damages of the coated gold can introduce line artifacts and fixed pattern noises. Therefore, an applied segmentation approach must cope with different types of artifacts. Figure 1 shows example images gathered with the PAMONO sensor containing different characteristics of artifacts. The intensities and occurring types can change for each experiment and also during one recording. Since tests with particles involve high material costs, the availability of the related images is low. In contrast, reference images showing only background and artifacts can be provided more efficiently. This property and the occurrence of various artifacts make the data acquired with the PAMONO sensor a well-suited example for evaluating our approach.

## 4. Methods

For increasing the robustness against artifacts in the analysis of sensor images, we formally introduce our method. We assume an image $I_{D_{j},t}\in {\left[0,1\right]}^{X_{D_{j}}\times Y_{D_{j}}}$ at a discrete timestep *t* originating from a data stream $D_{j}$ from the set of all image streams D to be composed of different signals in an additive signal model

(1) $$I_{D_{j},t}\left(x,y\right)=P_{D_{j},t}\left(x,y\right)+B_{D_{j},t}\left(x,y\right)+C_{D_{j},t}\left(x,y\right)+U_{D_{j},t}\left(x,y\right)$$

for $x\in \left[1,\ldots ,X_{D_{j}}\right],y\in \left[1,\ldots ,Y_{D_{j}}\right]$. The signal consists of a particle signal $P_{D_{j},t}$, a background $B_{D_{j},t}$, which is constant for all positions (x,y) within a single image, a correlated artifact signal $C_{D_{j},t}$, and uncorrelated artifacts $U_{D_{j},t}$. Both artifact components can contain values outside of [0,1]. For this work, we use images $I_{D_{j},t}$, which are already preprocessed with a sliding window method presented in previous work [56]. This preprocessing enhances the visibility of particle signals using temporal information for each image pixel and a dynamic contrast enhancement afterward. Figure 1 shows example images $I_{D_{j},t}$ for different datasets $D_{j}$ and timesteps *t* where $C_{D_{j},t}$ predominates with different artifact characteristics in each image. The goal here is to highlight all image positions containing a particle. Therefore, we want to find a function

(2) $$f:{\left[0,1\right]}^{X_{D_{j}}\times Y_{D_{j}}}\to {\left[0,1\right]}^{X_{D_{j}}\times Y_{D_{j}}}$$

to realize a semantic segmentation [75] to learn a mapping

(3) $$M_{D_{j},t}\left(x,y\right)=\left\{\begin{array}{cc} 1 & \mathrm{if}\;P_{D_{j},t}\left(x,y\right)>0\\ 0 & \mathrm{otherwise} \end{array}\right.$$

from images $I_{D_{j},t}$ onto a binary segmentation map. In order for *f* to achieve good results on a multitude of different datasets $D_{j}$, a broad set of artifacts has to be handled. Our approach expands a low-artifact data basis by augmenting the training data with additional artifacts. We make use of datasets $F_{k}\in F$ without particles of interest so that a contained image can be written as

(4) $$I_{F_{k},t}^{\left(\mathrm{artifact}\right)}\left(x,y\right)=B_{F_{k},t}\left(x,y\right)+C_{F_{k},t}\left(x,y\right)+U_{F_{k},t}\left(x,y\right).$$

Such images can be created without the need for test objects and serve as a basis for learning realistic characteristics of artifact patterns.

Having identified that wave-like artifacts are a factor that can heavily disturb detection methods, we also developed a method to generate wave-like artifacts directly to prepare the trained network towards being robust against possible correlated artifacts. This method serves as a basis for comparison to the presented GAN-based approach.

### 4.1. Artifact Overlays Based on Synthetic Artifacts

**Figure 2:**
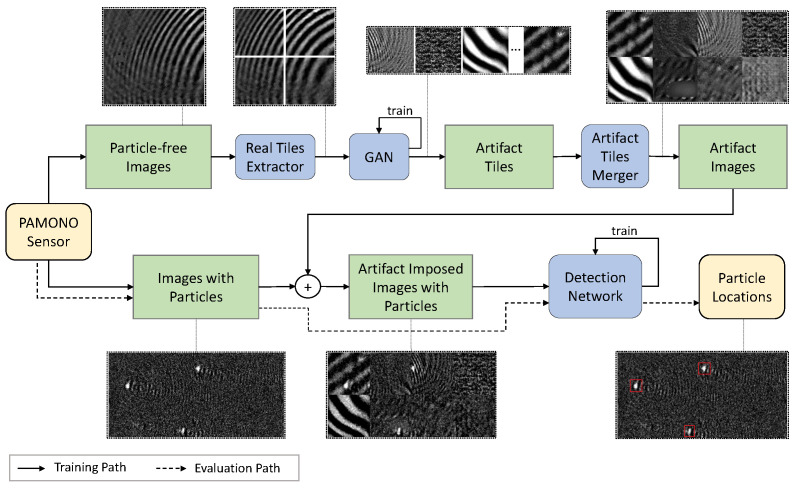
Schematic representation of overlaying training images with generative adversarial network (GAN)-generated artifacts from composite tiles. The PAMONO sensor is used to record samples without particles of interest (**upper part**) and samples including such particles (**lower part**) for the training process. The trained detection model is then used to search for particles in images where their presence is unknown. Dashed arrows show the path of images in the evaluation process, while solid arrows represent the path of images in the training process. The images in dotted boxes visualize the single steps by examples. The yellow boxes illustrate start and end of the pipeline, green boxes represent data and blue boxes mark algorithms.

From an abstract perspective, we overlay an image containing object signals of interest with a composite synthetic noise signal to optimize a segmentation model. Figure 2 shows an overview of this procedure. The upper part of the system shows the learning of artifact characteristics from images without object signals. Tiles are extracted from a recorded image and used for training a GAN. The GAN learns to generate new tiles, which are then combined into an artifact image. The lower part shows the overlay of a recording with a composition of generated artifact tiles.

In detail, we augment each training image $I_{D_{j},t}$ with structured artifacts $C^{\left(\mathrm{overlay}\right)}$ and uncorrelated artifacts $U^{\left(\mathrm{overlay}\right)}$. We combine both types to a single artifact signal

(5) $$A^{\left(\mathrm{overlay}\right)}\left(x,y\right)=C^{\left(\mathrm{overlay}\right)}\left(x,y\right)+U^{\left(\mathrm{overlay}\right)}\left(x,y\right)$$

and use it to create an augmented image

(6) $$I_{D_{j},t}^{\left(\mathrm{augment}\right)}\left(x,y\right)=I_{D_{j},t}\left(x,y\right)+A^{\left(\mathrm{overlay}\right)}\left(x,y\right).$$

**Figure 3:**
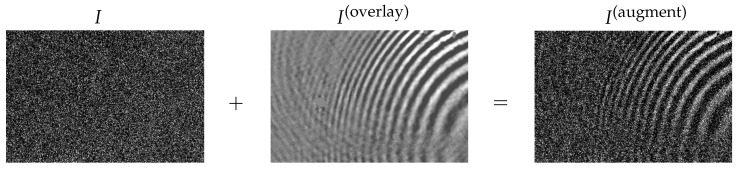
Example combination of artifacts according to Equation (6). A training image *I* with little correlated artifacts is augmented with wave artifacts $A^{\left(overlay\right)}$ to expand the present artifact patterns. The combination of artifacts is noted as $I^{\left(augment\right)}$.

Figure 3 shows an example of such an overlay.

In order to extract artifact signals from an image, we solve the assumed signal model of Equation (4) for artifact components

(7) $$A_{F_{k},t}\left(x,y\right)=I_{F_{k},t}\left(x,y\right)-B_{F_{k},t}\left(x,y\right).$$

Since we are only interested in the contained artifact signals, we use images without particle signals. Therefore, the only remaining unknown signal is the constant background signal. We assume that the artifact and noise signals are zero-centered. Consequently, we approximate the background as the mean intensity value

(8) $$\mu_{F_{k},t}=\frac{1}{X_{F_{k}}\;\cdot \;Y_{F_{k}}}\sum_{x=1}^{X_{F_{k}}}\sum_{y=1}^{Y_{F_{k}}}I_{F_{k},t}\left(x,y\right)$$

of the full image. The artifact signal $S_{F_{k},t}^{\left(\mathrm{overlay}\right)}$ can be formulated as

(9) $$A_{F_{k},t}^{\left(\mathrm{overlay}\right)}\left(x,y\right)=I_{F_{k},t}\left(x,y\right)-\mu_{F_{k},t}\;$$

for further use as an overlay. With these artifacts, the original images from a dataset $D_{j}$ can be augmented according to Equation (6).

Despite the reduced costs of producing images without involving particles for real artifact tiles, the available images are still limited. In order to have access to an unlimited stream of new and distinct artifacts, we propose the synthetic generation of new images $I_{F_{k},t}$. With this, we can provide an arbitrary number of synthetic but realistic-looking artifact patterns. Currently, the state-of-the-art method for image synthesis are generative adversarial networks (GANs) [58]. GANs use a generator model *G* to mimic the distribution of a set of real images optimized with feedback from a discriminator model *D*. The discriminator is optimized to distinguish between real and synthetic images. As the input for training the GAN, we use real images from a dataset $F_{k}$. In this work, we employ StyleGAN2-ADA [58], which is specifically designed for optimization with limited data. After optimizing the generative network, the generator function is used to create an arbitrary number of new artifact images.

The generated artifacts can be smaller than the original image $I_{D_{j},t}$. In this case, larger artifact images can be composed of multiple smaller ones. A set of artifact tiles

(10) $$A^{(\mathrm{overlays})}=\left\{A_{0}^{\left(\mathrm{overlay}\right)},\cdots ,A_{\left\lceil \frac{X_{D_{j}}}{v}\right\rceil \;\cdot \;\left\lceil \frac{Y_{D_{j}}}{w}\right\rceil }^{\left(\mathrm{overlay}\right)}\right\}$$

is generated where each artifact tile $A_{k}^{\left(\mathrm{overlay}\right)}$ is extracted from a synthetically generated image $I_{k}^{\left(\mathrm{overlay}\right)}$ with side lengths *v* and *w*. The tiles are then composed to a single artifact

(11) $$A^{\left(\mathrm{composed}\right)}\left(x,y\right)=A_{\left\lfloor \frac{x}{v}\right\rfloor +\left\lceil \frac{X_{D_{j}}}{v}\right\rceil \;\cdot \;\left\lfloor \frac{y}{w}\right\rfloor }^{\left(\mathrm{overlays}\right)}\left(x\;\mathrm{mod}\;v,y\;\mathrm{mod}\;w\right)$$

which has the needed size. For each training image, a new set $A^{\left(\mathrm{overlays}\right)}$ is dynamically generated.

### 4.2. Real Artifacts as Overlays

For a direct comparison, we apply real artifacts directly to the training images instead of applying synthetic artifacts. To create overlays from recorded data directly, we modify the set of artifacts $A^{(\mathrm{overlays})}$ to not originate from the GAN but from random cutouts from real images. We make use of non-annotated images which do not contain signals of objects of interest but are still affected by artifacts. Unlike in the GAN-based approach, the available data is directly limited by the original set of input images. This allows a meaningful comparison of the effects of learned artifacts with the direct utilization of real artifacts.

### 4.3. Procedurally Generated Artifact Signals

We present another approach for generating artifact patterns which is based on the procedural generation of artifacts in an attempt to simulate real artifacts in the form of imperfect waves superimposed over an image. In our observations, we found sine waves to be suitable approximations for actually recorded artifacts. These calculations are rules-based and can be varied using random parameter values.

Given an image *I* with side lengths *X* and *Y*, $n_{w}$ waves are generated and added to this image for training. For a single sine wave centered around point = $c_{w}=\left(c_{w_{x}},c_{w_{y}}\right)$, we determine the amplitude

(12) $$h\left(x,y,c_{w},\sigma ,\omega \right)=\sin\left(d\left(x,y,c_{w}\right)\;\cdot \;\sigma +\omega \right)$$

at every image position x∈[1,…,X],y∈[1,…,Y] using a frequency parameter σ, a phase shift ω and a distance

(13) $$d\left(x,y,c_{w}\right)=\sqrt{{\left(c_{w_{x}}-x\right)}^{2}+{\left(c_{w_{y}}-y\right)}^{2}}\;.$$

We observed that the intensities of waves in an image are often not constant over the entire surface, so a term

(14) $$e\left(x,y,c_{f},\beta \right)=1-\frac{d{\left(x,y,c_{f}\right)}^{\beta }}{\max(\left\{g{\left(a,b,c_{f}\right)}^{\beta }\;|\;1\leq a\leq X,1\leq b\leq Y\right\})}$$

is included to add a fading effect starting from an independant center point $c_{f}$ from which the intensity decreases with a rate β∈[0,1]. This term is applied to the original wave function *h* to receive a single fading wave

(15) $$h^{\prime }\left(x,y,c_{w},c_{f},\sigma ,\omega ,\beta \right)=h\left(x,y,c_{w},\sigma ,\omega \right)\;\cdot \;e\left(x,y,c_{f},\beta \right).$$

**Figure 4:**
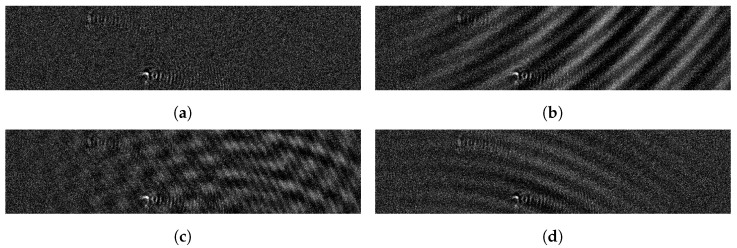
An (**a**) input image and (**b**–**d**) examples of randomly generated wave artifacts.

Finally, all $n_{w}$ waves are composed and added to the image *I* to simulate a combination

(16) $$I^{\left(\mathrm{waves}\right)}\left(x,y,C_{w},C_{f},S,W,B,\gamma \right)=I\left(x,y\right)+\frac{\left(\sum_{i=1}^{n_{w}}h^{\prime }\left(x,y,c_{w_{i}},c_{f_{i}},\sigma_{i},\omega_{i},\beta_{i}\right)\right)\;\cdot \;\gamma }{n_{w}}$$

of different vanishing waves by using sets of wave centers $C_{w}=\left\{c_{w_{1}},\ldots ,c_{w_{n_{w}}}\right\}$, fade centers $C_{f}=\left\{c_{f_{1}},\cdots ,c_{f_{n_{w}}}\right\}$, frequency parameters $S=\{\sigma_{1},\cdots ,\sigma_{n_{w}}\}$, phase shifts $W=\{\omega_{1},\cdots ,\omega_{n_{w}}\}$, and fade rates $B=\{\beta_{1},\cdots ,\beta_{n_{w}}\}$. The influence of the waves in the resulting image is controlled via the wave strength factor γ. The parameter values for each wave are randomly chosen from a restricted interval. Figure 4 shows examples of randomly generated wave artifacts added to a low artifact image. The resulting wave artifacts approximate the visual appearance of real artifacts with parameters drawn from a manually defined interval.

Although it is possible to find fitting intervals that result in a distribution similar to real artifacts, a procedural generation of artifacts requires the manual definition of the generating function and manual tuning to the artifact characteristics at hand.

## 5. Experiments

We evaluate our GAN-based method by applying it to image streams recorded with the PAMONO sensor that is described in Section 3. Individual image streams show different artifacts, so it offers a well-suited opportunity to evaluate this approach. The goal is to find a model that solves the segmentation of particles, as formulated in Section 4. Particles should be easily distinguishable from other image parts in the resulting segmentation, so we employ a blob detection based on Difference of Gaussians (DoG) [76] features for particle detection. To focus the evaluation on the augmentations only, we employ a plain 5-layer U-Net [77] with 16 filters in the first layer. We make no changes to this architecture during our experiments and only conduct changes for the data itself. In this way, we can evaluate the effectiveness of our proposed approach and compare it directly to the other introduced methods. This provides a concrete implementation of the abstract detection network shown in Figure 2. The different approaches are compared to each other based on correctly detected nanoparticles.

We utilize the dice loss [78] in combination with the Adam [79] optimizer to train the U-Net. An initial learning rate of $3\times 10^{-5}$ is halved after every 15 epochs with no improvement in the dice loss for designated validation datasets. We end the training after 30 epochs with no improvement. For this work, 23 annotated image streams containing particles of interest provide 30,782 images in total. Only one of these datasets with low intensities of artifacts and well visible particle regions containing 500 images is used for training. We employ five datasets as validation data. The remaining datasets are used as test data after the training is completed.

Due to the preprocessing, each particle contained in the image streams can be seen not only on one but on several frames. We connect the particle locations on individual images to traces afterward. This means that sufficiently overlapping regions on consecutive frames are combined to one particle, which is especially important for counting particles to determine the viral load in a sample [56].

For measuring run times, an Nvidia Geforce GTX 1080 GPU is used. Random cutouts with side lengths of 128 pixels from 1157 images originating from a single reference image stream are used for training the GAN. About 5 GB of video memory are allocated. Using a batch size of 16, around 38 h are needed for training a StyleGAN2-ADA network consisting of a generator part with $23\times 10^{6}$ parameters and a discriminator part with $24\times 10^{6}$ parameters. The training times for the U-Net lie between 90 min with no augmentation and up to 360 min for the GAN-based augmentations. For better comparability, the same dataset for training the GAN is used for overlaying images with real artifacts.

To compare the GAN-approach also to a direct and simple augmentation we apply a variation of image sizes relative to the sizes of particle regions in the samples. For each training dataset $D_{j}$ the median surface $s_{D_{j},\mathrm{med}}$ of annotated particle regions in the dataset is calculated to determine the overall minimum size

(17) $$s_{\min}=\min\left(s_{D_{j},\mathrm{med}}\;|\;D_{j}\in D\right)$$

and the maximum size $s_{\max}$ analogously. The median operator is used to determine sizes within a dataset in order to compensate for possible outliers caused by manual annotation. By restricting the random factor $F_{D_{j}}$ used to scale both sides of an image separately to

(18) $$f_{D_{j}}=\left[\sqrt{\frac{s_{\min}}{s_{D_{j},\mathrm{med}}}},\sqrt{\frac{s_{\max}}{s_{D_{j},\mathrm{med}}}}\right]$$

for a dataset $D_{j}$, the scaled images cover the range of particle sizes seen as plausible based on the available annotations. In each training step the side lengths *u* and *v* of a training image $I_{D_{j},t}$ are scaled by a factor $f_{d}\in F_{d}$ to $u\;\cdot \;f_{D_{j}}$ and $v\;\cdot \;f_{D_{j}}$. Since this approach presents a simple strategy that has proven useful in combination with more complex approaches in preliminary tests, it is also applied in the case of procedural wave generation, real artifact overlays, and GAN-based overlays.

For each evaluated configuration of augmentations, we consider two measures. The first measure is the F1-score [80]

(19) $$F_{1}=\frac{2\;\cdot \;precision\;\cdot \;recall}{precision+recall}=\frac{tp}{tp+0.5\;\cdot \;(fp+fn)}$$

of particle traces which uses the number of true positives (tp), false positives (fp), and false negatives (fn) to indicate the extent to which the predicted traces and the annotations match. A predicted trace is seen as matching if its bounding box overlaps significantly with the box of an annotated trace. As two overlapping predictions can both be seen as true positives when overlapping with one annotated trace, this measure focuses on the accuracy of particle locations instead of matching trace counts.

The second measure is the count exactness [56]

(20) $$e\left(n_{a},n_{p}\right)=1-\frac{|n_{a}-n_{p}|}{\max(n_{a},n_{p})}$$

which compares the number of predicted traces $n_{p}$ with the number of annotated traces $n_{a}$. As the count exactness does not consider where the single traces are located, false positives and false negatives can misleadingly balance each other out. Nevertheless, it is a simple and practice-oriented measure that is especially of interest in real use case scenarios, where an expert can interpret this information based on domain knowledge. In PAMONO sensor data, the determined particle count could be compared to expected concentrations of virus particles related to an infection of interest.

We execute each training configuration three times to reduce the effect of outliers. The model with the median F1-score is selected for evaluating all presented metrics. We compare the proposed GAN-based approach in Table 2 with the alternatives based on F1-scores and count exactness values related to particle traces. The results vary heavily for different datasets depending on the intensities and prevalent types of artifacts in the contained images. Therefore, we also show results for datasets split into different groups of artifacts. A comparison broken down by the qualitative type of dominant artifacts is given in Table 3.

We also compare the approaches using the binary distinction between samples containing particles of interest and samples free of them. The exact particle counts and locations are less relevant here. Instead, an effective separation between these two groups is sought, for which a low number of false positives in particle-free samples is essential. Results for samples of this type are conducted in Table 4, and the counts of predicted particles per image are compared for models trained with the different approaches. For this purpose, 12 particle-free datasets with 10,384 images in total showing diverse artifact types and intensities are analyzed.

## 6. Discussion

Aiming at high robustness of a learned segmentation against imaging artifacts, our approach using GANs to generate synthetic artifacts shows to be the most effective. Compared to the version with no augmentation, as shown in Table 2, this approach yields improvements of 22% in the F1-score, 26% in the average count exactness, and even greater improvements in the related minimum values. Table 3 shows that the results improve more with stronger visible artifacts and correlation within these. The GAN approach increases the F1-score by 63% and the average count exactness by 61% for datasets with wave-like artifacts. In the task of searching for particles in particle-free samples, this approach improves the average number of false positive particle traces from 0.87 to 0.02 per image, with the dataset performing the worst, only having 0.05 false-positive traces per image.

Comparing the GAN-based approach with extracting artifacts directly from images, the span between the worst and best values is smaller. The augmentation by superimposing wave artifacts based on a hand-crafted, procedural function is approximately on par with the augmentation with real artifacts when considering average scores. However, minimum values show a slight improvement, which indicates greater stability of the detection after the appropriate training. The real and the procedurally generated artifacts improve the F1-score by 14% compared to the training without augmentations. This shows that the model benefits significantly from augmentation with correlated artifacts. Viewing the results in Table 2, it is noticeable that direct augmentation, representing the random size augmentation based on the particle sizes present in the training dataset, does not improve the F1-score and the count exactness for datasets containing particles. Compared to the basic version without augmentation, there is even a slight deterioration in the F1-score. If the evaluation is expanded to the datasets not containing particles of interest, the impression is different. Table 4 shows that the average rate of false positives per image can be reduced by 94.5% by just applying direct size augmentations.

All in all, the augmentation by overlaying with artifacts generated by our GAN-based approach achieves the most significant improvements, both in the average and minimum values. The increase of the minimum values can be seen as better robustness against artifacts that do not occur in the training data. At the same time, despite the increased training time, the advantage of not having to define and adjust a function description by hand can be noted. This shows that the GAN-based generation of artifact images for data augmentation can be a worthwhile improvement to classic augmentations in image analysis. This holds especially when the exact artifact patterns can only be described with great effort, for example, when the application environment of the used sensor changes frequently while a lack of training data makes the determination difficult.

## 7. Outlook

Since our approach showed to be capable of increasing the robustness of a spatial learning system against image artifacts, the exploitation of temporal correlations can be investigated. In image data streams, objects of interest and artifact patterns are time-dependent in most cases, so generating time-consistent artifacts could further improve the results for a downstream task. It needs to be considered that, while the complexity of the generation task increases, fewer spatiotemporal training samples can be formed from a set of images. Despite the potential problems, evaluating a generation approach incorporating the temporal dimension can further increase the robustness of a downstream, spatiotemporal image analysis. Our approach demonstrates that it mitigates the effects of artifacts in images of the PAMONO sensor. Further work should evaluate this method for images from other sensors. The approach has the potential to be applied to other sensors with little customization.

## Figures and Tables

**Table 1:** Overview of common artifact types in sensor images, their properties, sources, and examples for algorithmic reduction methods. Correlated artifacts are also called structured noise, and uncorrelated artifacts are called unstructured. Temporally changing artifacts can vary in each frame.

Artifact Type	Correlated		Temporally Changing		Artifact Sources	Algorithmic Methods for Reduction
	Yes	No	Yes	No		
Shot noise [4,6]		•	•		environment	classic filters (e.g., median filter) [7], bilateral filtering [8], neural networks [9], wavelet/Fourier filtering [10]
Readout noise [6]		•	•		electronics	
Thermal noise [11]		•	•		environment, electronics	
Salt and pepper noise [7]		•	•		electronics	
Random telegraph noise [4]		•	•		electronics	
Temporal contrast/ brightness inconsistencies [12]	•		•		electronics, environment, software	homomorphic filtering [13], stabilization algorithms [14], temporal filtering [12], neural networks [15]
Line, stripe, wave and ring artifacts [16,17]	•		•		electronics, environment, optics	wavelet/Fourier filtering [10], spatial filtering [16], neural networks [18]
Compression artifacts [19]	•		•		software	bilateral filtering [8], fuzzy filtering [20] neural networks [19,21,22,23]
Projective distortions [24]	•			•	optics	model-based calculations [25], neural networks [26,27]
Out-of-focus effects [28,29]	•			•	optics	morphological filtering [30], neural networks [31,32]
Fixed pattern noise [33,34]	•			•	electronics, environment, optics	reference imaging [33], neural networks [35]
Aliasing [36]	•		•		software	anti-aliasing algorithms [36], neural networks [37]
Rolling shutter effects [38]	•		•		electronics	neural networks [39]

**Table 2:** F1-score and count exactness values measured after training with the presented augmentation methods. The best results are written in bold.

	**Metric**	**Average** **F1-Score**	**Minimum** **F1-Score**	**Average** **Count Exactness**	**Minimum** **Count Exactness**
**Augmentation**					
No augmentation		0.62	0.07	0.53	0.03
Only direct augmentation		0.60	0.06	0.54	0.03
Procedurally generated waves		0.76	0.29	0.67	0.16
Real artifacts		0.76	0.15	0.68	0.08
GAN-generated artifacts		**0.84**	**0.55**	**0.79**	**0.48**

**Table 3:** F1-score (F1) and count exactness (CE) values for samples containing particles of interest after training with different augmentation methods broken down by dominant artifact types. The best results are written in bold.

Data Group		Highly Visible Particles		Stronger Noises or Temporal Inconsistencies		Wave-like Artifacts	
	Metric	F1	CE	F1	CE	F1	CE
Augmentation							
No augmentation		0.85	0.74	0.51	0.44	0.10	0.05
Only direct augmentation		0.88	0.80	0.40	0.36	0.18	0.09
Procedurally generated waves		0.89	0.82	0.70	0.58	0.49	0.37
Real artifacts		0.91	0.84	0.67	0.57	0.46	0.40
GAN-generated artifacts		**0.92**	**0.88**	**0.78**	**0.71**	**0.73**	**0.66**

**Table 4:** Number of falsely predicted particles (FP) per image for datasets containing no particles of interest measured after training with different augmentation methods. The best results are written in bold.

	**Metric**	**Average** **FP per Image**	**Maximum** **FP per Image**
**Augmentation**			
No augmentation		0.87	6.55
Only direct augmentation		0.05	0.36
Procedurally generated waves		0.06	0.30
Real tiles artifacts		0.10	0.38
GAN-generated artifacts		**0.02**	**0.05**

## Data Availability

Example datasets with samples containing particles of interest and samples without such particles can be found at https://graphics-data.cs.tu-dortmund.de/docs/publications/panomo/ (accessed on 5 October 2021).
